# Long non-coding RNA signature for predicting gastric cancer survival based on genomic instability

**DOI:** 10.18632/aging.205336

**Published:** 2023-12-20

**Authors:** Jialing Zhang, Liang Chen, Wei Wei, Fei Mao

**Affiliations:** 1 Department of Gastroenterology, The Affiliated Huaian No. 1 People’s Hospital of Nanjing Medical University, Huaian 223300, Jiangsu, People’s Republic of China; 2 Department of Hepatobiliary and Pancreatic Surgery, Conversion Therapy Center for Hepatobiliary and Pancreatic Tumors, First Hospital of Jiaxing, Affiliated Hospital of Jiaxing University, Jiaxing 314000, Zhejiang, P.R. China; 3 Department of Anesthesiology and Pain Research Center, The First Hospital of Jiaxing or The Affiliated Hospital of Jiaxing University, Jiaxing 314000, Zhejiang, China; 4 Department of Urology, The Affiliated Huaian No. 1 People’s Hospital of Nanjing Medical University, Huaian 223300, Jiangsu, People’s Republic of China

**Keywords:** genomic instability, gastric cancer, lncRNA, prognosis, immune checkpoint

## Abstract

Background: Gastric cancer is a prevalent type of tumor with a poor prognosis. Given the high occurrence of genomic instability in gastric cancer, it is essential to investigate the prognostic significance of genes associated with genomic instability in this disease.

Methods: We identified genomic instability-related lncRNAs (GInLncRNAs) by analyzing somatic mutation and transcriptome profiles. We evaluated co-expression and enrichment using various analyses, including univariate COX analysis and LASSO regression. Based on these findings, we established an lncRNA signature associated with genomic instability, which we subsequently assessed for prognostic value, immune cell and checkpoint analysis, drug sensitivity, and external validation. Finally, PCR assay was used to verify the expression of key lncRNAs.

Results: Our study resulted in the establishment of a seven-lncRNA prognostic signature, including PTENP1-AS, LINC00163, RP11-169F17.1, C8ORF87, RP11-389G6.3, LINCO1210, and RP11-115H13.1. This signature exhibited independent prognostic value and was associated with specific immune cells and checkpoints in gastric cancer. Additionally, the model was correlated with somatic mutation and several chemotherapeutic drugs. We further confirmed the prognostic value of LINC00163, which was included in our model, in an independent dataset. Our model demonstrated superior performance compared to other models. PCR showed that LINC00163 was significantly up-regulated in 4 adjacent normal tissues compared with the GC tissues.

Conclusions: Our study resulted in the establishment of a seven-lncRNA signature associated with genomic instability, which demonstrated robust prognostic value in predicting the prognosis of gastric cancer. The signature also identified potential chemotherapeutic drugs, making it a valuable tool for clinical decision-making and medication use.

## INTRODUCTION

T.H. Morgan’s discovery of genetic mutations in fruit flies over 100 years ago was a groundbreaking achievement that greatly advanced the fields of genetics and cancer biology [1]. Genetic mutation is a frequent occurrence in organisms, and the accumulation of mutations is a driving force in tumor formation [2]. Genomic instability, a hallmark of tumors, promotes mutation and enables tumors to adapt to their environment through evolutionary mechanisms, leading to increased tolerance and drug resistance [3]. Gene mutations linked to genomic instability include those affecting DNA repair, tumor suppressor genes, and proto-oncogenes [4]. Additionally, lncRNAs have been implicated in the development of genomic instability [5, 6]. Despite this knowledge, the mutation landscape, mechanisms of action, and prognostic value of these genomic instability-related genes and lncRNAs at different tumor stages remain unclear.

Non-coding RNAs, despite not participating in protein-coding, play a crucial role in gene regulation and signaling pathway regulation [7]. LncRNAs, a class of non-coding RNAs with nucleotides greater than 200, have been shown in numerous studies to be significantly related to tumors [8]. They are involved in various biological processes, including gene transcription, translation, and post-translational modification, and constitute a vast regulatory network [9]. Despite the increasing number of studies on lncRNAs, the current knowledge is still insufficient.

Gastric cancer, a major global health burden, ranked as the fifth most common malignancy worldwide in 2018, with the third-highest fatality rate [10]. The current consensus is that gastric cancer is not a single disease but an individualized disease [11], owing to its high heterogeneity [12]. This heterogeneity also leads to the diversification of treatment methods, including surgical treatment, chemotherapy, targeted therapy, immunotherapy, among others [12]. Although the prognosis of gastric cancer is improving, the survival rate of advanced and refractory cases remains poor [13]. Identifying gastric cancer patients at high risk with poor prognosis and establishing specific treatment plans for them remains a challenging problem.

Genomic instability is common in gastric cancer due to its high heterogeneity [14], and the network of lncRNAs may interact with genomic instability, allowing for the construction of a genomic instability-related lncRNA signature in this disease [15]. In gastric cancer, genomic instability is a hallmark of the disease and plays a critical role in tumor initiation and progression [16]. Genomic instability refers to a high rate of genetic alterations, including DNA mutations, chromosomal rearrangements, and copy number variations, that can lead to altered gene expression and dysregulated cellular functions [16]. Several lncRNAs have been identified as key players in cancer pathogenesis by regulating genomic instability [17, 18]. For example, the lncRNA HOX transcript antisense RNA (HOTAIR) has been shown to promote gastric cancer progression by inducing chromatin remodeling and altering gene expression patterns [19]. Another lncRNA, metastasis-associated lung adenocarcinoma transcript 1 (MALAT1), has been implicated in gastric cancer metastasis by modulating the expression of genes involved in cell migration and invasion [20]. Furthermore, emerging evidence suggests that lncRNAs can also regulate DNA damage response pathways and contribute to genomic instability in gastric cancer. For instance, the lncRNA TUG1 has been reported to promote DNA damage and enhance the sensitivity of gastric cancer cells to radiation therapy [21].

In this study, we identified lncRNAs associated with genomic instability in gastric cancer and established their significant relation to prognosis. These prognostic lncRNAs were used to construct a risk-scoring model for gastric cancer patients, providing new insight for prognosis guidance and targeted treatment of this disease.

## MATERIALS AND METHODS

### Data download and processing

We downloaded somatic mutation data (MuTect2 Variant mutation and Masking data) of 437 gastric cancer samples, transcriptome data of 407 samples (including STAD-Counts and STAD-FPKM), and clinical data of 348 gastric cancer patients from The Cancer Genome Atlas (TCGA) database [22]. We also obtained data of 300 gastric cancer patients with clinical data and transcriptional information from the GEO database (data set GSE62254) [23]. For subsequent analysis, we used the COUNT data type for differential analysis, and the FPKM data type was converted to the TPM (transcripts per kilobase of exon model per million mapped reads) data type [24]. All expression data were transformed by log2 for further analysis [25]. After excluding patients without clinical data or with 0 survival time, we obtained a final cohort of 348 gastric cancer patients with both clinical information and expression data. We randomly divided these patients into a training group and a test group, as well as a combined dataset (Table 1). PCR belongs to the second half (validation part), using samples from patients of The Affiliated Huaian No. 1 People’s Hospital of Nanjing Medical University.

**Table 1:** The clinical data of 348 gastric patients in the training group, testing group and the total dataset.

**Covariates**	**Type**	**Training**	**Testing**	**Total**	**p-value**
Gender	Female	61(34.66%)	62(36.05%)	123(34.66%)	0.874
Gender	Male	115(65.34%)	110(63.95%)	225(64.66%)	
Age	<65	77(43.75%)	71(41.28%)	148(42.53%	0.623
Age	>=65	95(53.98%)	99(57.56%)	194(55.75%)	
Age	Unknown	4(2.27%)	2(1.16%)	6(1.72%)	
T stage	T1&T2	48(27.27%)	42(24.42%)	90(25.86%)	0.5081
T stage	T3&T4	127(72.16%)	127(73.84%)	254(72.99%)	
T stage	Unknown	127(72.16%)	3(1.74%)	4(1.15%)	
M stage	MO	158(89.77%)	153(88.95%)	311(89.37%)	0.2969
M stage	M1	13(7.39%)	9(5.23%)	22(6.32%)	
M stage	Unknown	5(2.84%)	10(5.81%)	15(4.31%)	
N stage	N0	47(26.7%)	56(32.56%)	103(29.6%)	
N stage	N1-3	121(68.75%)	113(65.70%)	234(67.24%)	0.1933
N stage	Unknown	8(4.55%)	3(1.74%)	11(3.16%)	
Stage	StageI&II	73(41.48%)	83(48.26%)	156(44.83%)	0.4282
Stage	StageIII&IV	96(54.55%)	82(47.67%)	178(51.15%)	
Stage	Unknown	7(3.98%)	7(4.07%)	14(4.02%)	
Status	Alive	104(59.09%)	99(57.56%)	203(58.33%)	0.8562
status	dead	72(40.91%)	73(42.44%)	145(41.67%)	

### LncRNAs associated with genomic instability were obtained

We utilized the R-package “maftools” to analyze mutation data of gastric cancer samples, calculating the total number of mutations in each sample. Patients were then sorted based on their mutation load, defining the top 25% as genome-stable (GS-like) patients and the bottom 25% as genome-unstable (GU-like) patients. Differences in total mutation load and the expression of UB1LN4, a gene related to genomic instability, were examined between the two groups. The lncRNA expression data for the two groups were obtained by matching transcriptome data, and the “DEseq2” package was used for differential analysis of lncRNAs between the two groups. Genomic instability-related lncRNAs (GlnLncRNAs) were identified (|logFC| > 2 and *p* < 0.05), and genetic variations were visualized through heatmaps and volcano plots, with the five most up-regulated and down-regulated lncRNAs labeled. Spearman correlation analysis was performed between the 101 differential lncRNAs and coding proteins, with the top 10 mRNAs most associated with each lncRNA being selected. The lncRNA-mRNA network was then mapped to illustrate their relationships. Additionally, functional and pathway enrichment analysis of differential genes between the genome-stable and genome-unstable groups was conducted through the “clusterProfiler” package.

### Building a prognostic model

We finally obtained 13 prognosis-related lncRNAs by the univariate Cox analysis. In the training group, we then performed Lasso regression analysis on these 13 genes to construct a prognostic model. We then summed up the risk score by multiplying the amount of gene expression in each patient’s model by the corresponding correlation coefficient in the training set, validation set, and the entire data set. After obtaining a risk score for each patient, we divided the patients in the three datasets into low-risk and high-risk groups based on the median risk value of the patients in the training group. The risk score was calculated with the formula as follows: $\text{Riskscore}=\sum_{i=0}^{n}\text{coefi}*\text{X}i$. The “coefi” and “X_i_” represent the coefficient and expression level of each prognostic lncRNAs.

### Evaluation of the model

We assessed the relationship between risk score and prognosis in the training group, test group, and entire dataset, as well as the accuracy of predicting 5-year survival using the area under the ROC curve. Differences in the number of mutations and the expression of UB1LN4, a gene associated with genomic instability, were examined between the high-risk group or the genomically stable group and the genomically unstable group. We analyzed whether the model had the same value across different populations, such as gender, age, and early and late-stage tumors. Univariate and multivariate Cox analyses were used to evaluate whether the model could be used as an independent prognostic indicator for gastric cancer patients in these three datasets. We created a dynamic nomogram representing patient A in the dataset to evaluate the 1-, 3-, and 5-year mortality of patients with gastric cancer. Additionally, calibration curves were plotted to evaluate the accuracy of the model in predicting outcomes at 1, 3, and 5 years.

### Correlation between the model and immune cells and checkpoint

We downloaded seven types of algorithms from the TIMER database (http://timer.cistrome.org/) to assess immune infiltration in each patient. We analyzed the expression of immune cells in the high and low-risk groups, identified cells with differential expression (*p* < 0.05), and developed a heatmap. We also studied the expression of immune checkpoint genes between the high and low-risk groups, extracting genes with different expressions (*p* < 0.05) and creating a boxplot. Additionally, we used the DREIMT database (http://www.dreimt.org/) to further analyze the correlation between the model and immune cells. We input 80 down-regulated genes and the first 199 up-regulated genes in ascending order of *p*-value into the website for verification.

### Model to predict potential drug candidates for gastric cancer

We utilized the “pRRophetic” package and the expression matrix of gastric cancer patients to predict the minimum drug inhibition concentration (IC50) of drugs in gastric cancer patients. Based on the model, we identified drugs that could become candidates for the treatment of gastric cancer. Additionally, we used the DREIMT database (http://www.dreimt.org/) to predict potential drugs related to the risk for gastric cancer by inputting 80 differential genes in the high and low-risk groups and the first 199 up-regulated genes in ascending order of *p*-value into the website.

### Explore the pathways associated with the model

We downloaded the Hallmarks data set from the Gene Set Enrichment Analysis database (http://www.gsea-msigdb.org/gsea/msigdb/index.jsp). We then used the Gene Set Variation Analysis (GSVA) algorithm to calculate the enrichment fraction of a special gene set in the Hallmarks data set, based on expression data from the high-risk group of patients with gastric cancer. We analyzed the differences between the high and low-risk groups using LIMMA (*p*< 0.05), and obtained 42 significantly enriched pathways, which were displayed in the form of a histogram.

### External dataset validation and model comparison

We attempted to further assess the accuracy of the signature by exploring data sets associated with gastric cancer from the GEO and ICGC databases. However, we could not find any data set that included all of the lncRNAs in the signature. Therefore, we chose the GSE62254 data set with clinical data to explore the role of LINC00163 in gastric cancer. Additionally, we compared the 5-year accuracy under the curve of our signature with other models.

### PCR to verify the expression of LINC00163 in GC

PCR experiments were conducted to compare gene expression levels between 4 pairs of GC and Para-GC tissues from The Affiliated Huaian No. 1 People’s Hospital. Total RNA was isolated from the tissues using the TRIzol reagent. cDNA was synthesized using the PrimeScript™ RT reagent kit. Real-time PCR was performed using the SYBR Green Real-time PCR Master Mix on an Applied Biosystems QuantStudio 7 Flex Real-time PCR System. The relative gene expression levels were calculated using the 2^−ΔΔCt^ method, with GAPDH as the internal reference gene. The primer sequence of LINC00163 is Forward: GAGCAAGCTCTAGCTCTCGG; Reverse: AGAGCTTTGGGAAGACACCG. GAPDH Forward: AATGGGCAGCCGTTAGGAAA; Reverse: GCCCAATACGACCAAATCAGAG. All experiments were performed in triplicate, and the data were analyzed using GraphPad Prism software.

### Statistical analysis

Chip-squared and Rank sum test (Wilcoxtest) test was used to assess the difference in categorical data between different groups as well as datasets. The version of R software was 4.0.5. The statistical significance was defined with the two-tailed *p<0.05*.

## RESULTS

### The flow chart

**Figure 1:**
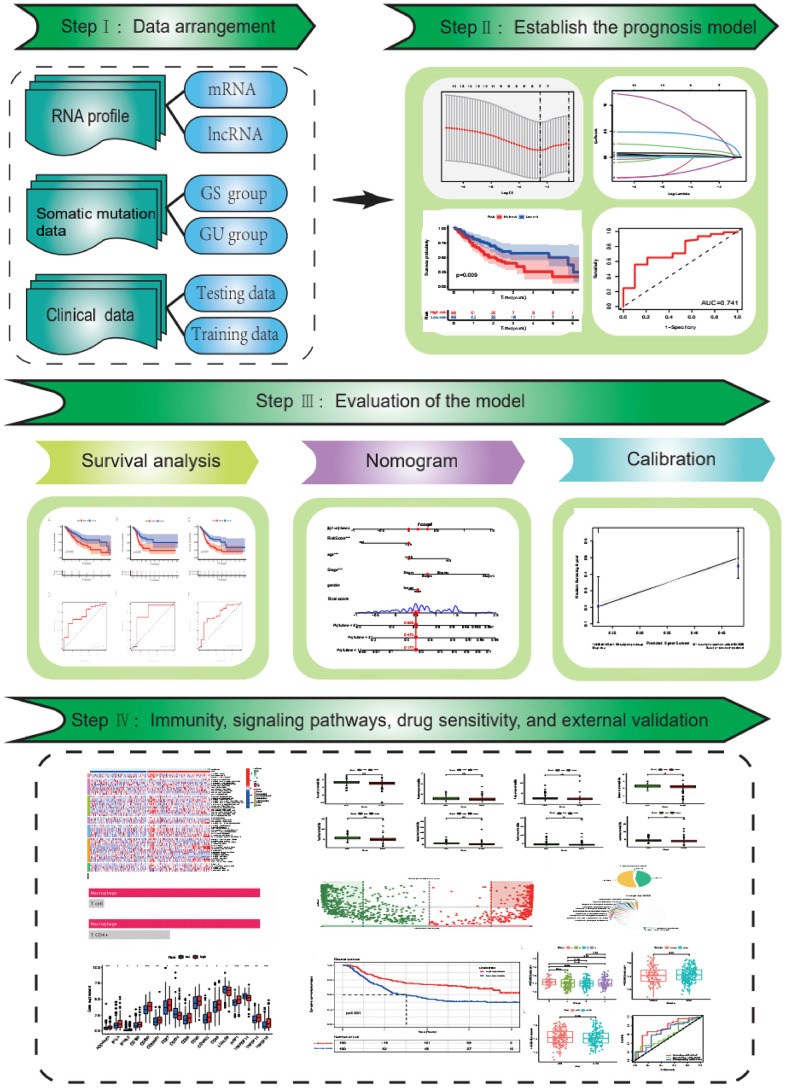
The flow chart.

Our research process is summarized in Figure 1.

### Data download and processing

We randomly divided the 348 patients with gastric cancer into the training group and the test group, as well as the combined dataset. The specific information is shown in Table 1, and there were no significant differences in clinical data between the training group and the test group (*p* > 0.05).

### LncRNAs associated with genomic instability were obtained

**Figure 2:**
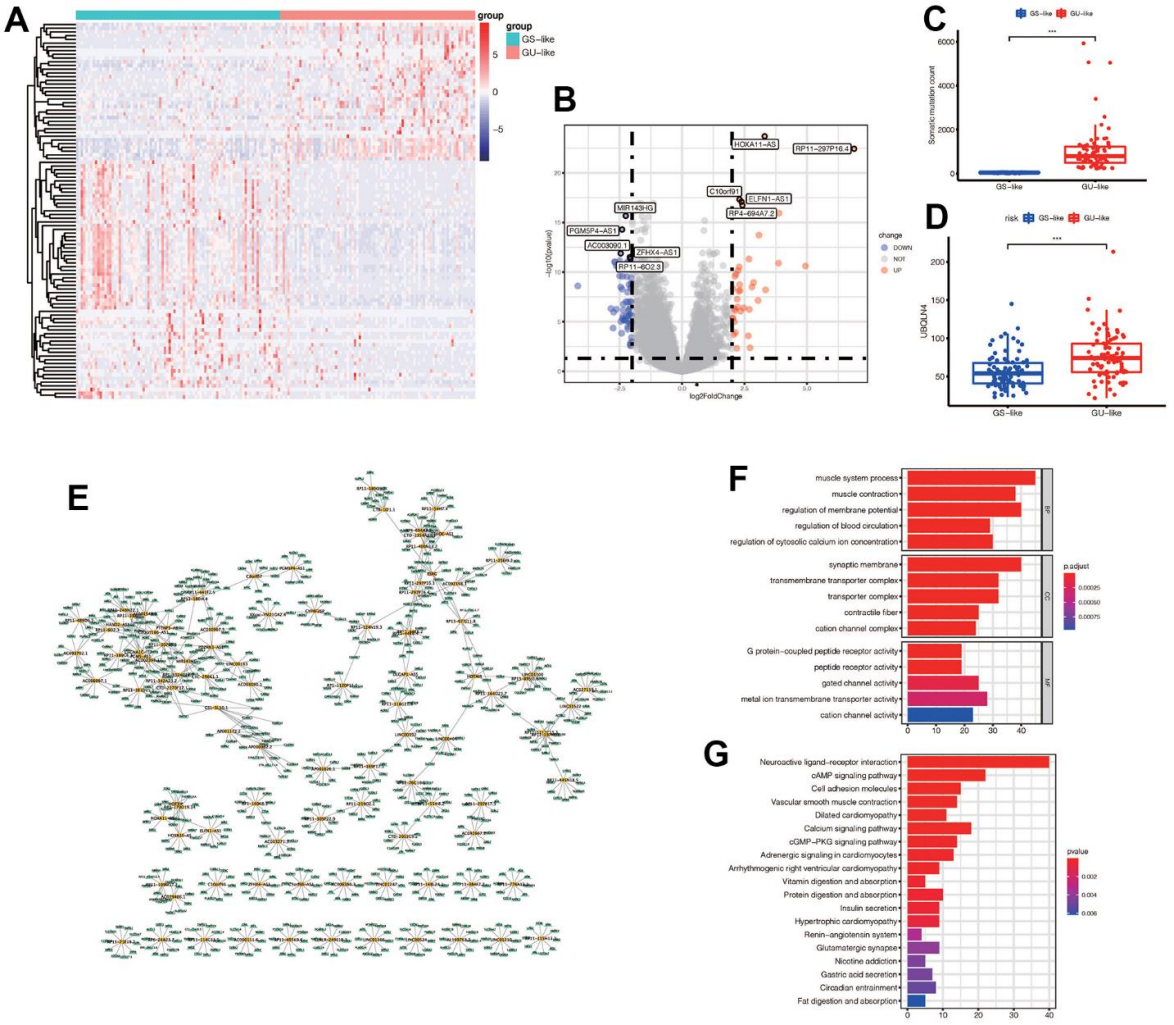
(**A**) Expression of differential genes in GS-like group and GU-like group. The darker the red, the higher the expression level, and the darker the blue, the lower the expression level. (**B**) The display of differential genes in the volcano map: the top five most up-regulated lncRNAs and the top five most down-regulated lncRNAs were labeled respectively. The blue indicated down-regulated lnRNA, and the red indicated up-regulated lncRNAs. (**C**) The number of somatic mutations in GS-like group and Gu-like group was different (*** *p* <0.001). (**D**) The expression of UBQLN4 gene was different between the GS-like group and the Gu-like group (*** *p* <0.001). (**E**) The differentially expressed lncRNAs and the top 10 most related mRNAs formed a co-expression network, with yellow representing lncRNAs and green representing mRNAs. (**F**) GO enrichment analysis in GS-like group and GU-like group. The top five most significantly enriched functions were extracted from BP, CC and MF (*p*<0.05). (**G**) KEGG enrichment.

We obtained 101 genomic instability-related lncRNAs (GlnLncRNAs) with | logFC | > 2 and *p*< 0.05. We created a heatmap and volcano plot to visualize the differential expression of the GlnLncRNAs, and labeled the top five most significantly up-regulated and down-regulated lncRNAs. The top five down-regulated lncRNAs were MR143HG, PGM5P4-AS1, AC003090.1, ZFHX4-AS1, and the up-regulated lncRNAs were HOXA11-AS, RP11-297P16.4, CQ0ORF91, ELFN1-AS1, RP4-694A7.2. Additionally, we found that the somatic mutation count and the expression of UBQLN4 were different in the GS-like and GU-like groups, and were up-regulated in the GU-like group (*p*< 0.05) (Figure 2C–2D). We also identified that the lncRNAs and mRNAs formed a network and interacted with each other in gastric cancer (Figure 2E). Furthermore, we conducted functional enrichment analysis of the differentially expressed genes between the GS-like and GU-like groups, and found that they were mainly associated with receptor activity, G protein-coupled peptide receptor activity, cAMP signaling pathway, cell adhesion molecules, calcium signaling pathway, and cGMP-PKG signaling pathway, which are involved in the tumorigenesis and development of gastric cancer (Figure 2F–2G).

### Construction of the prognostic model

**Figure 3:**
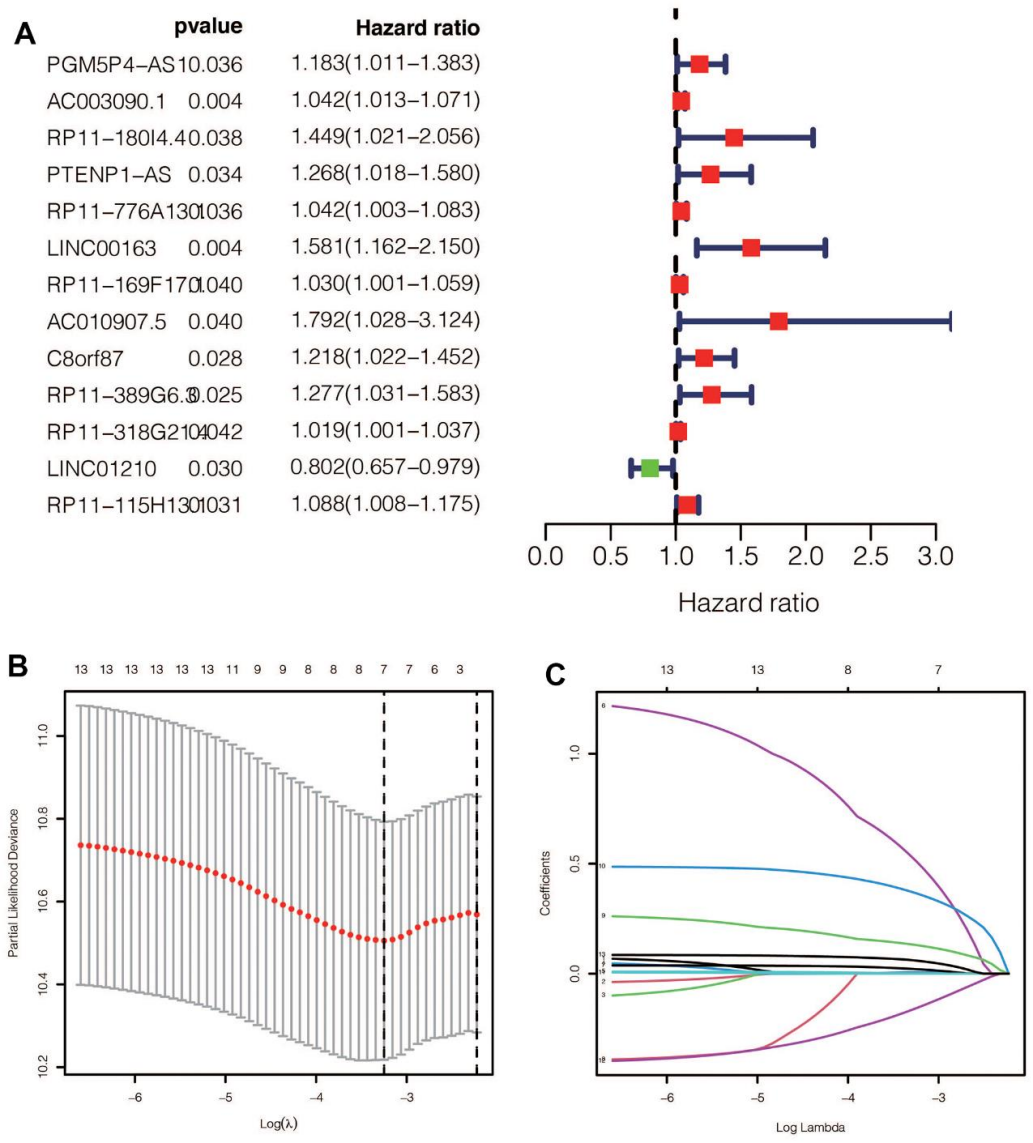
(**A**) After univariate Cox regression, 11 prognostic related ginlncRNAs were obtained, of which 10 were deleterious genes and one was protective gene. (**B**, **C**) Lasso regression results showed that when the best lambda value was 0.01149079, the following curve tended to be stable, and 7 lncRNAs were selected for the model construction.

After performing univariable Cox regression, we identified 13 lncRNAs that were significantly associated with prognosis (Figure 3A). Subsequently, we performed Lasso regression analysis on these 13 lncRNAs in the training group, with an optimal Lambda value of 0.01149079 (Figure 3B, 3C). Ultimately, we identified seven GlnLncRNAs, including PTENP1-AS, LINC00163, RP11-169F17.1, C8ORF87, RP11-389G6.3, LINCO1210, and RP11-115H13.1 (Table 2). Using the weights of these seven lncRNAs in the training set, validation set, and combined dataset, we calculated the risk score for each patient. Patients were then divided into high-risk and low-risk groups based on the median score in each dataset. The risk score calculation was as follows: risk score = PTENP1-AS * 0.00635 + LINC00163 * 0.51230 + RP11-169F17.1 * 0.02056 + C8ORF87 * 0.13023 + RP11-389G6.3 * 0.36579 + LINCO1210 * (-0.15732) + RP11-115H13.1 * 0.05948.

**Table 2:** The detail information of the seven GlnlncRNAs in the signature.

**GlnlncRNAs**	**Description**	**Coefficient**
PTENP1-AS	PTENP1 Antisense RNA	0.00635
LINC00163	Long Intergenic Non-Protein Coding RNA 163	0.51230
RP11-169F17.1	Long Intergenic Non-Protein Coding RNA 2864	0.02056
C8ORF87	Chromosome 8 Open Reading Frame 87	0.13023
RP11-389G6.3	An RNA Gene affiliated with the lncRNA class	0.36579
LINCO1210	Long Intergenic Non-Protein Coding RNA 1210	-0.15732
RP11-115H13.1	Novel Transcript, Sense Intronic To TBL1Y	0.05948

### Evaluation of the model

**Figure 4:**
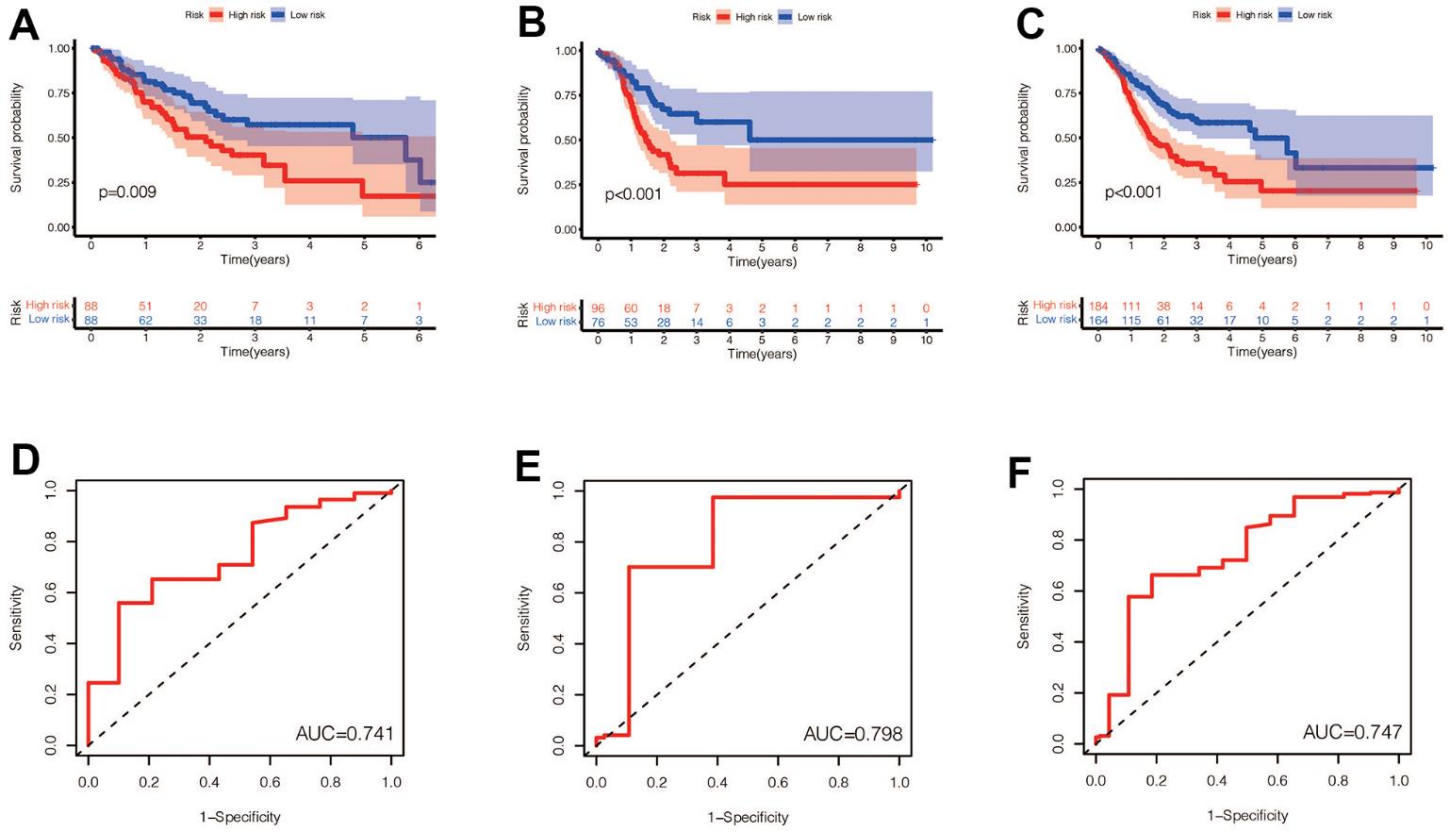
(**A**–**C**) Prognostic differences between the high-risk and low-risk groups were investigated in the training set, validation set, and the entire dataset. High risk was found to indicate poor prognosis in all three datasets (*p*<0.001). (**D**–**F**) In the training set, validation set and the entire set, the AUC values obtained by the prognostic model to predict the accuracy of the 5-year survival rate of patients were 0.741, 0.798 and 0.747, respectively.

**Figure 5:**
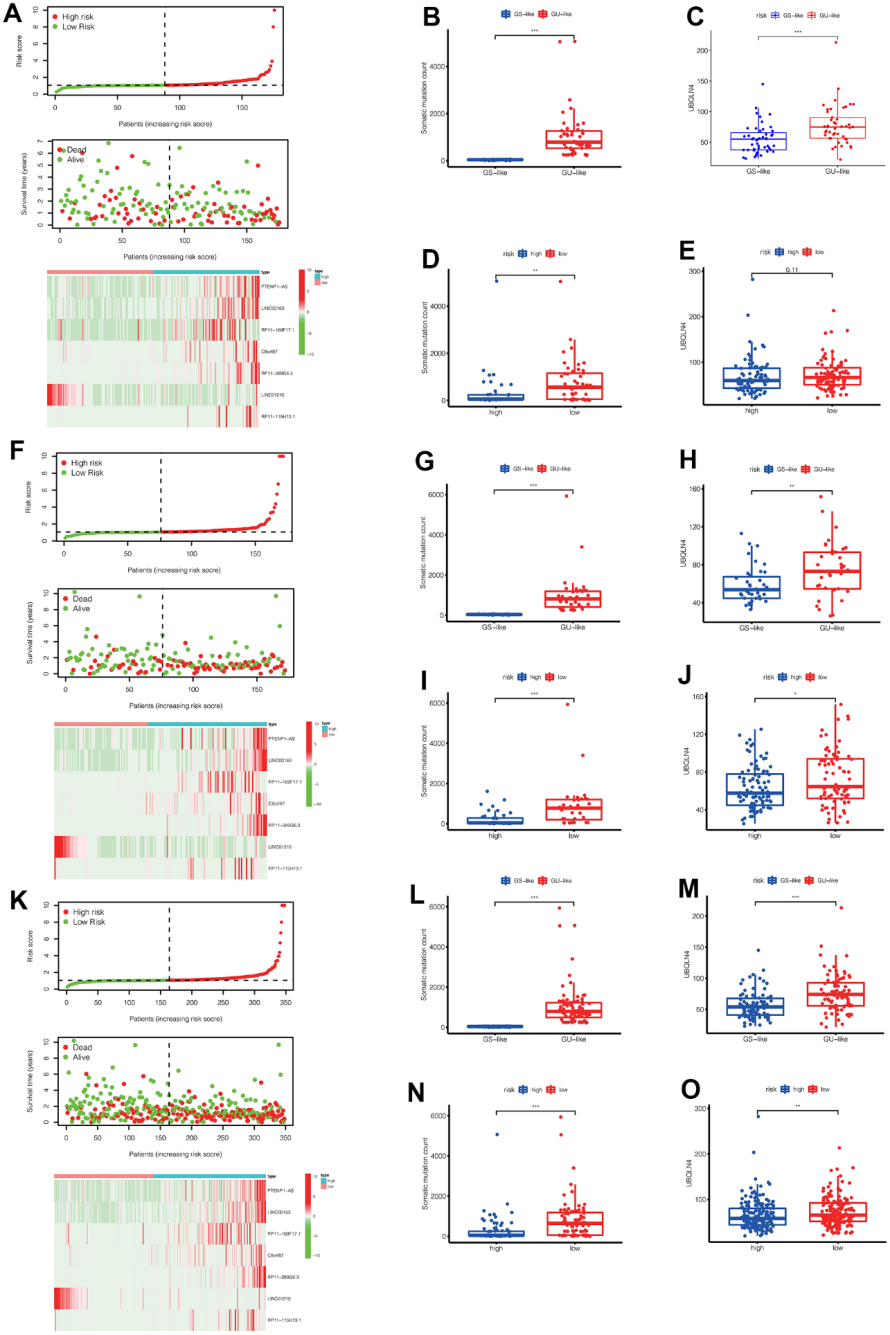
(**A**) In the training set, we can see the distribution of patient survival and death as the risk score increased, as well as the heat map of lncRNAs expression in the high-risk and low-risk groups. (**B**, **C**) In the training set, the number of somatic mutations and the expression of UBQLN4 gene were different between the GS-like group and the Gu-like group (** *p*<0.01, *** *p*<0.001). (**D**, **E**) In the training set, the number of somatic mutations and the expression of UBQLN4 gene were different between the high-risk group and the low-risk group (** *p*<0.01, *** *p*<0.001). (**F**) In the validation set, we can see the distribution of patient survival and death as the risk score increased, as well as the heat map of lncRNAs expression in the high-risk and low-risk groups. (**G**, **H**) In the validation set, the number of somatic mutations and the expression of UBQLN4 gene were different between the GS-like group and the Gu-like group (** *p* <0.01, *** *p*<0.001). (**I**, **J**) In the validation set, the number of somatic mutations and the expression of UBQLN4 gene were different between the high-risk group and the low-risk group (** *p* <0.01, *** *p* <0.001). (**K**) In the entire set, we can see the distribution of patient survival and death as the risk score increased, as well as the heat map of lncRNAs expression in the high-risk and low-risk groups. (**L**, **M**) In the entire set, the number of somatic mutations and the expression of UBQLN4 gene were different between the GS-like group and the Gu-like group (** *p*<0.01, *** *p* <0.001). (**N**, **O**) In the entire set, the number of somatic mutations and the expression of UBQLN4 gene were different between the high-risk group and the low-risk group (** *p* <0.01, ***p <0.001).

**Figure 6:**
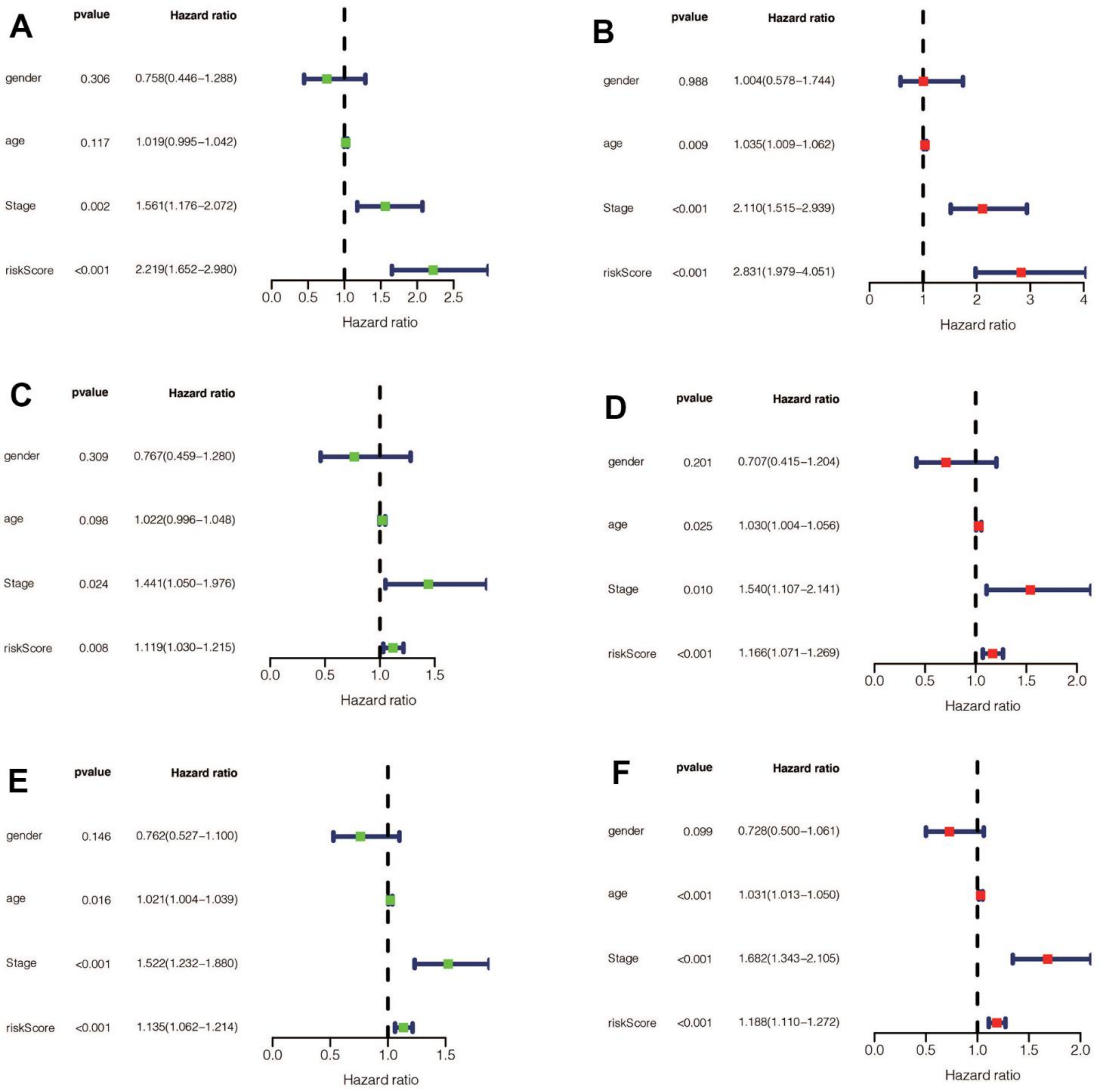
(**A**) Univariate Cox regression for risk-score, gender, age, and tumor stage in the training set found that risk score and stage were independent prognostic factors (*p*<0.05). (**B**) Multivariate Cox regression for risk-score, gender, age, and tumor stage in the training set found that risk score and stage were independent prognostic factors (*p*<0.05). (**C**) Univariate Cox regression for risk-score, gender, age, and tumor stage in the validation set found that risk-score and stage were independent prognostic factors (*p*<0.05). (**D**) Multivariate Cox regression for risk-score, gender, age, and tumor stage in the validation set found that risk score, age, and stage were independent prognostic factors (*p*<0.05). (**E**) Univariate Cox regression for risk-score, gender, age, and tumor stage in the entire set found that risk-score, age, and stage were independent prognostic factors (*p* <0.05). (**F**) Multivariate Cox regression for risk-score, gender, age, and tumor stage in the entire set found that risk score, age, and stage were independent prognostic factors (*p* <0.05).

**Figure 7:**
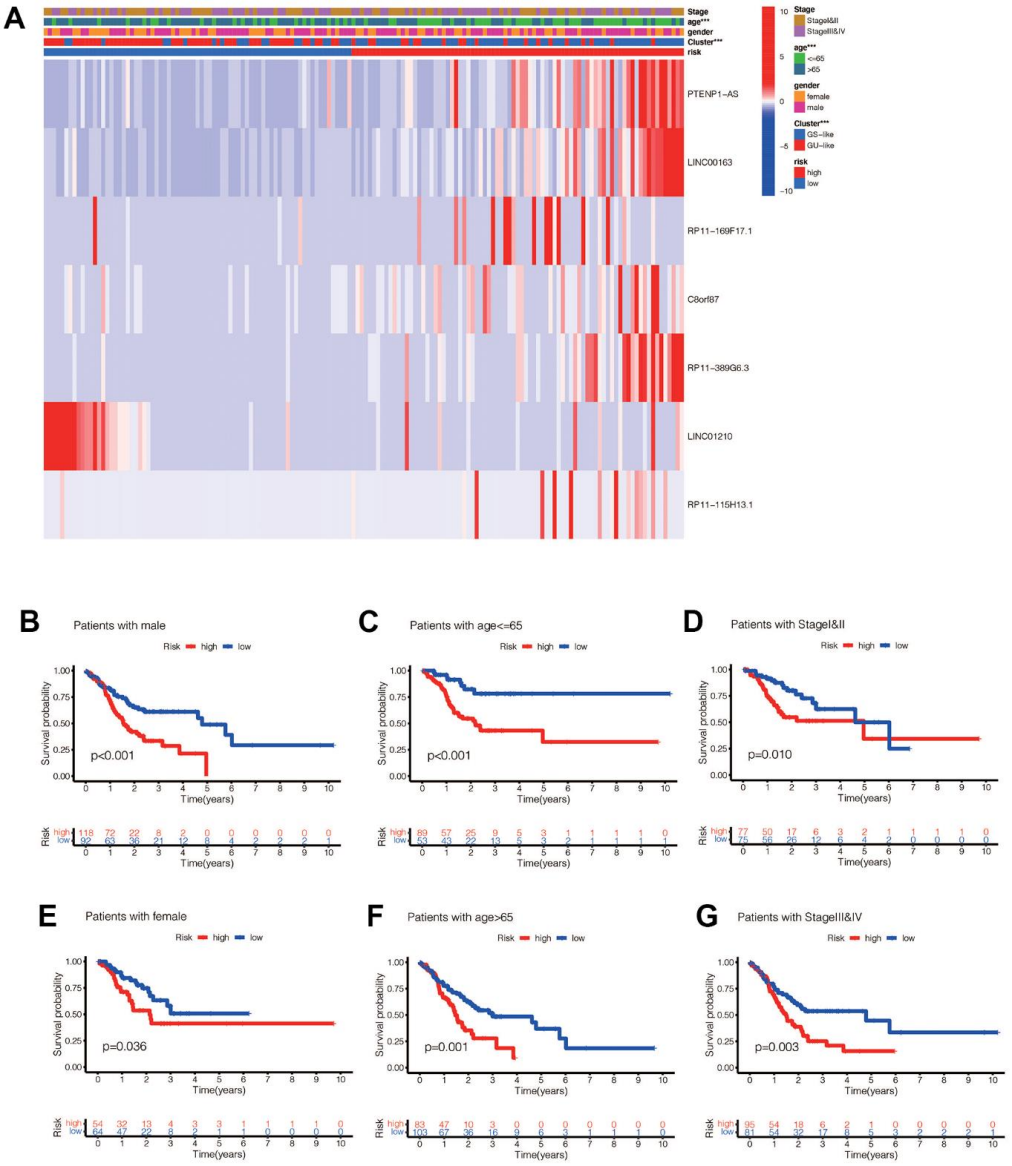
(**A**) Heat maps of the relationship between ginlncRNAs in high-risk group and low-risk group, GU-like group and GS-like group, tumor stage, age, and sex (* *p*<0.01, ** *p*<0.01, *** *p*<0.001). (**B**) The risk values of the constructed model were associated with poorer prognosis in male gastric cancer patients (*p*<0.05). (**C**) The risk value of the established model was associated with poor prognosis in patients with gastric cancer aged <=65 years (*p*<0.05). (**D**) The risk value of the constructed model was associated with poor prognosis in both early gastric cancer patients (*p*<0.05). (**E**) In female patients (*p*<0.05); (**F**) in aged >65 patients (*p*<0.05); (**G**) in late stage patients (*p*<0.05).

**Figure 8:**
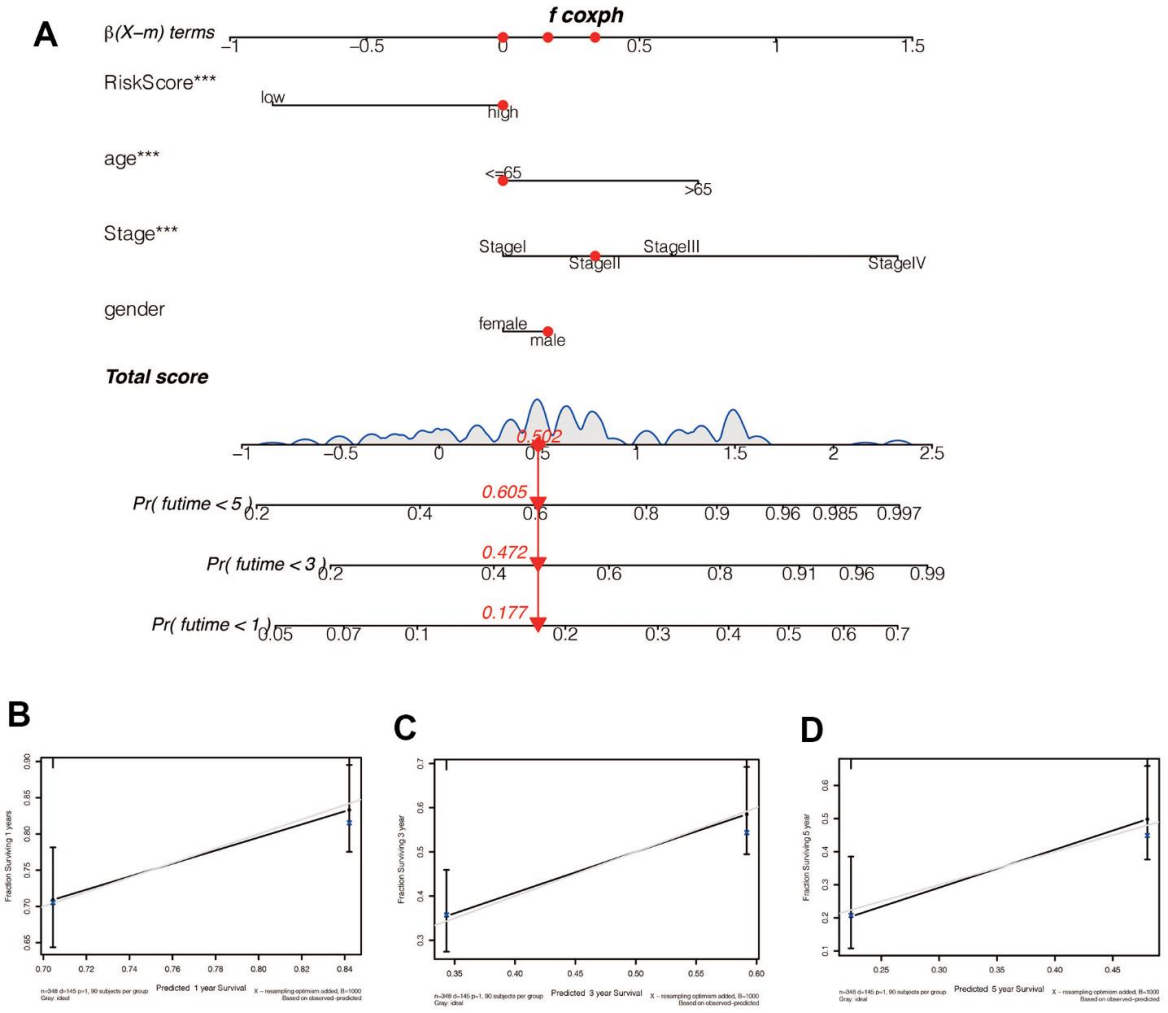
(**A**) A Nomogram for predicting prognosis based on risk value, age, tumor stage, and sex shows the clinical characteristics of the patient (TCGA-CG-571) and the predicted 1 -, 3 - and 5-year mortality of 0.177, 0.472, and 0.605, respectively. (**B**–**D**) The 1 -, 3 - and 5-year calibration curve constructed by risk value was used to evaluate the accuracy of prognosis prediction based on risk score, and the results were all satisfactory.

We performed a survival analysis of patients in the high-risk and low-risk groups. Outcomes were significantly worse in the high-risk group than in the low-risk group in the training set (Figure 4A, *p*=0.009), significantly worse in the validation set (Figure 4B, *p*<0.001), and similar results were obtained in the merge set (Figure 4C, *p* <0.001). The ROC curve was used to verify the accuracy of the survival analysis. The area under the curve (AUC) was 0.741 in the training set (Figure 4D), 0.798 in the validation set (Figure 4E), and 0.747 in the merge set (Figure 4F). As pictures shows (Figure 5A, 5F, 5K), LINC00163 was up-regulated in low-risk group, while the others were up-regulated in high-risk group. With the rising of risk score, the probability of death is up. We then analyzed the number of somatic mutations between the high-risk and low-risk groups, and between the Gu-like and GS-like groups. The results (Figure 5B, 5D, 5G, 5I, 5L, 5N) showed that in the three data sets, the number of somatic mutations was higher in the high-risk group or the Gu-like group, and the difference was statistically significant (*p*<0.05). The expression analysis of UBQLN4 also showed (Figure 5C, 5E, 5H, 5J, 5M, 5O) that the expression of UBQLN4 was mainly upregulated in the high-risk group compared with the low-risk group or the GU-like group comparing with the GS-like group (*p*<0.05). After univariate and multivariate Cox regressions of risk score and patient clinical characteristics (gender, age, stage) in three datasets, we found that risk score and stage were independent prognostic factors for patients (Figure 6A–6F, *p*<0.05). The heatmap (Figure 7A) showed the relationship between 7 lncRNAs and stage, age, sex, risk score, GS-like, and Gu-like, finding that there is a difference in GS-like and GU-like group as well as age group (*p*<0.001). Then, all patients were divided into two groups according to gender, age (greater than 65 years old, less than or equal to 65 years old), and stage respectively. The results showed that high-risk patients had a poor prognosis in groups by sex, age, and stage (Figure 7B–7G, *p*<0.05), which suggested that the signature had a similar result in different types of gastric patients. Dynamic Nomogram (Figure 8A) was used to predict the 1,3,5year overall survival of patients with gastric cancer and the calibration curve shows that the signature has a high prediction accuracy (Figure 8B–8D).

### Correlation between the model and immune cells

**Figure 9:**
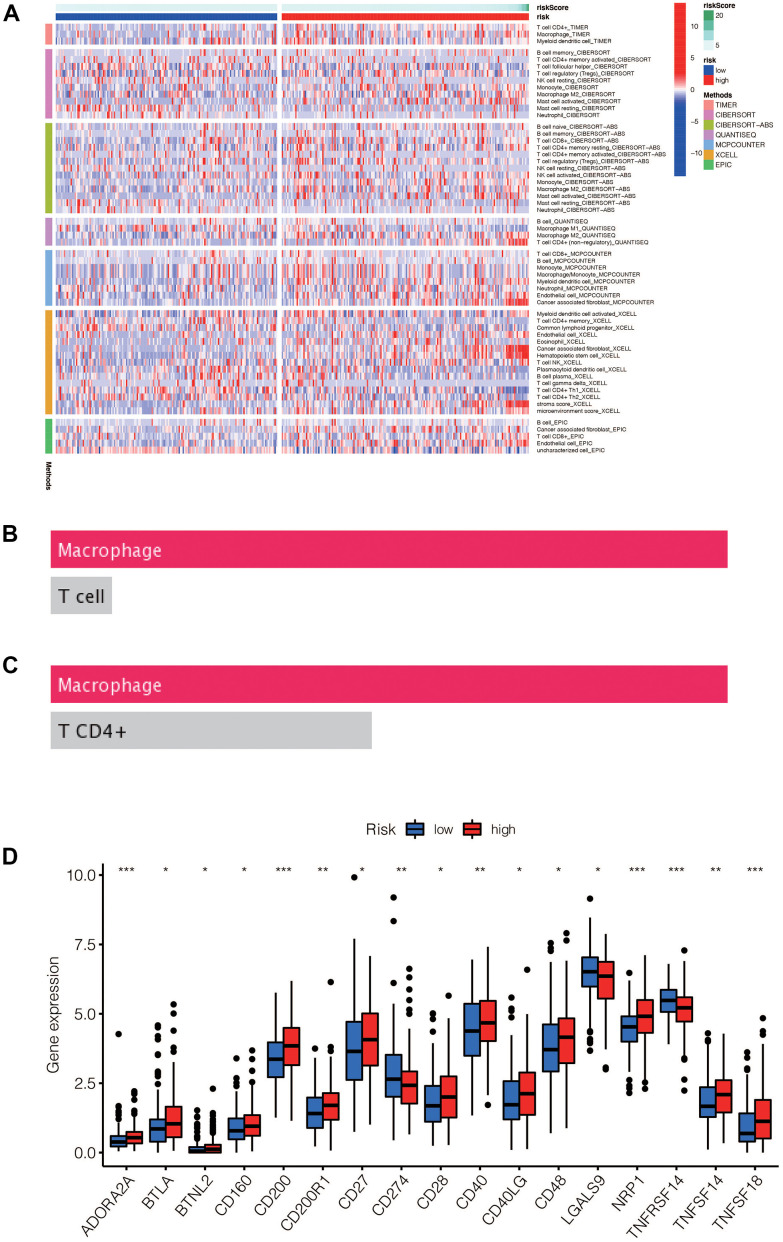
(**A**) Heat maps of immune cells that differ between the high-risk and low-risk groups. (**B**, **C**) Macrophage was the most different immune cells predicted by Dreimt database among the high-risk and low-risk groups (*p*<0.05). (**D**) Immune checkpoint genes that were differentially expressed in the high-risk and low-risk groups were selected and presented in a boxplot. (* *p*< 0.05, **p < 0.01, * * * *p*< 0.001).

The immune-related heatmap displayed the immune landscape based on high- and low-risk groups, where the infiltration level of various immune cells was obtained using different algorithms such as TIMER, CIBERSORT, QUANTISEQ. As shown in Figure 9A, there was a noticeable difference in the expression of immune cells between the two groups, with a trend of immune cells being up-regulated in the high-risk group compared to the low-risk group, such as macrophages, T cells CD4, and T cells CD8. To further verify the accuracy of this finding, we predicted the potential immune cells using the DREIMT database, which showed a similar result indicating that macrophages were highly associated with the risk groups (Figure 9B, 9C, *p*<0.05). We also analyzed the relationship between the high-risk and low-risk groups and immune checkpoint genes, where we found that the expression of immune checkpoint genes was mainly up-regulated in the high-risk group (Figure 9D, *p*<0.05).

### Potential drug candidates for gastric cancer

**Figure 10:**
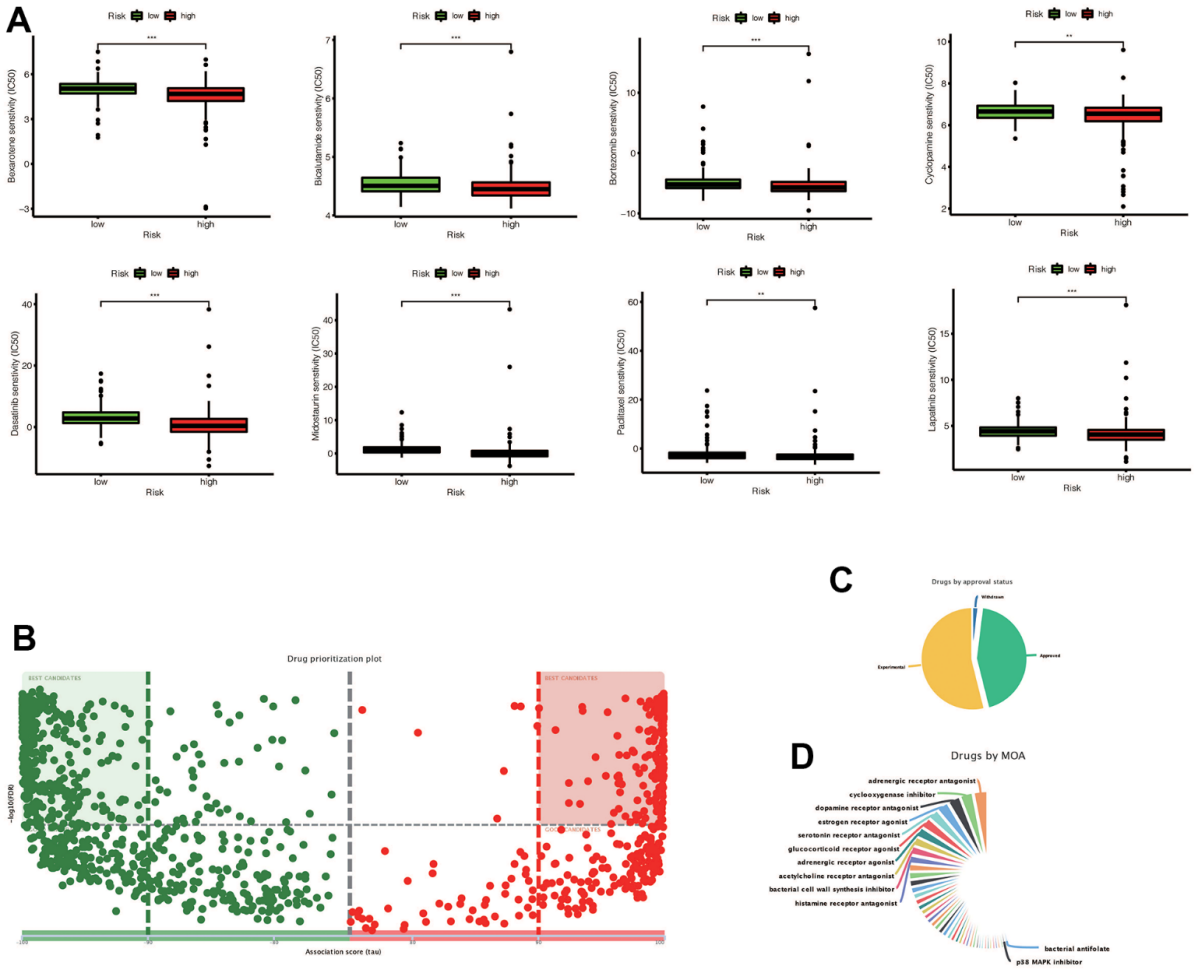
(**A**) Chemotherapy drugs with differential IC50 expression in the high-risk and low-risk groups predicted by the Prophetic package, and drugs with low IC50 expression in the high risk group were screened out (* * *p* < 0.01, ***p < 0.001). (**B**) The drugs predicted by DREMIT website are different in the high and low risk groups, in which the best candidates in the first and second quadrants mean the candidates with more reliable results, while the remaining drugs mean better candidates. (**C**) Classification of predicted chemotherapeutic drug validation levels. (**D**) Drug classification of predicted chemotherapeutic agents.

Our analysis showed that certain drugs, including Sunitinib and Shikonin, had lower IC50 concentrations in the high-risk group (Figure 10A). These findings suggest that patients in the high-risk group may be more sensitive to these drugs. In addition, using the DREIMT database, we predicted and identified candidate drugs for future treatment strategies (Figure 10B–10D). Our top 5 candidate drug types were adrenergic receptor antagonists, cyclooxygenase inhibitors, dopamine receptor antagonists, estrogen receptor agonists, and serotonin receptor antagonists. These results could inform personalized treatment approaches in the future.

### Exploration of the pathways associated with the model

**Figure 11:**
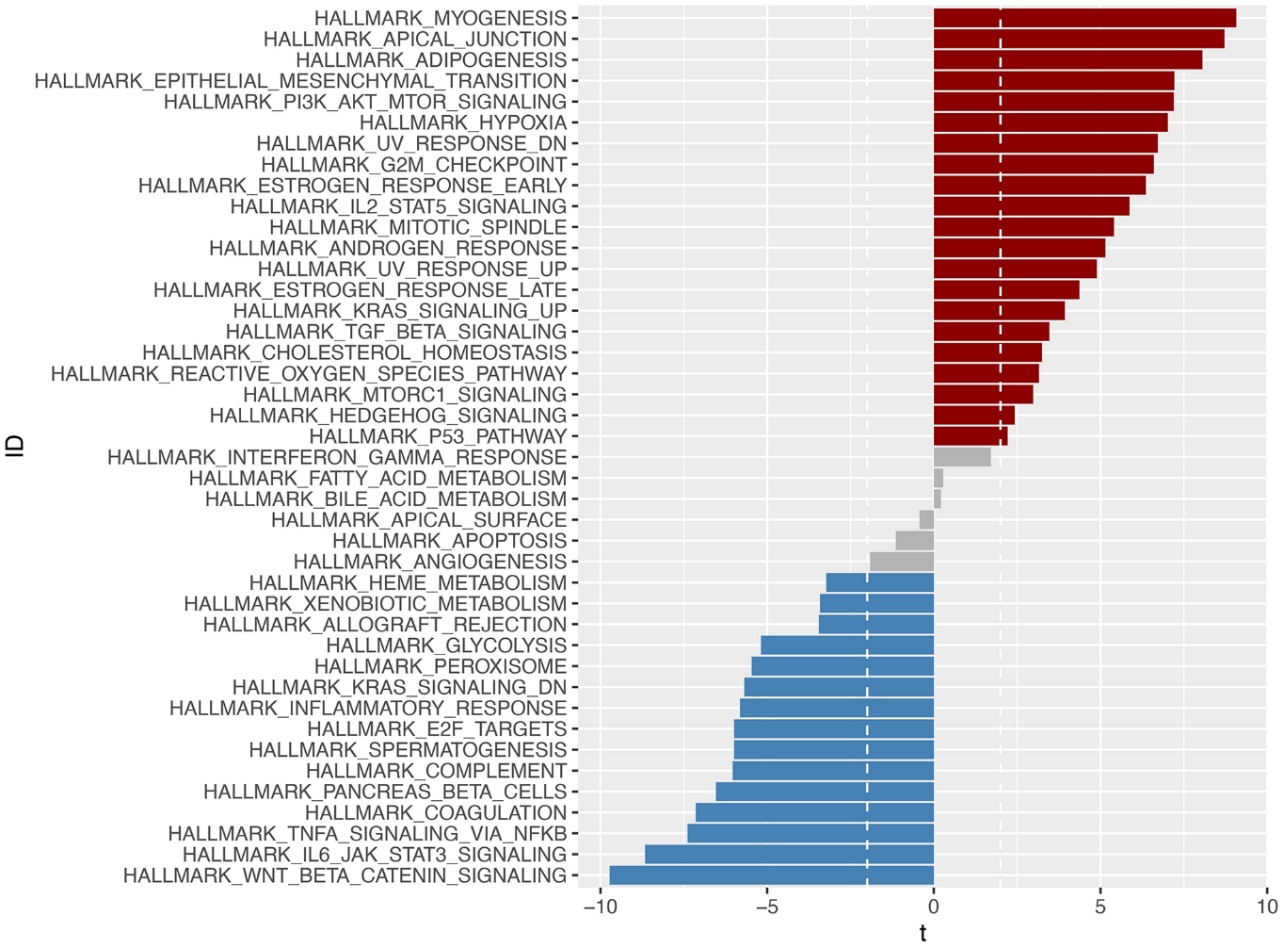
**Through GSVA analysis, significant enrichment pathways with differences in the high-risk and low-risk groups were obtained (*p* <0.05).** Pathways enriched in the high-risk group were marked in red, those enriched in the low-risk group were marked in blue, and those enriched in -2<t<2 were marked in gray.

We conducted a GSVA enrichment analysis and identified 42 significantly enriched pathways, as shown in Figure 11. This analysis allowed us to explore differences in signaling pathways between the high-risk and low-risk groups. Among the pathways associated with cancer, we found that the EMT signaling pathway, P13-AKT-TOR signaling pathway, and WNT-BETA-CATEIN signaling pathway were highly enriched. These pathways are known to play important roles in tumorigenesis and development in gastric cancer.

### External dataset validation and model comparison

**Figure 12:**
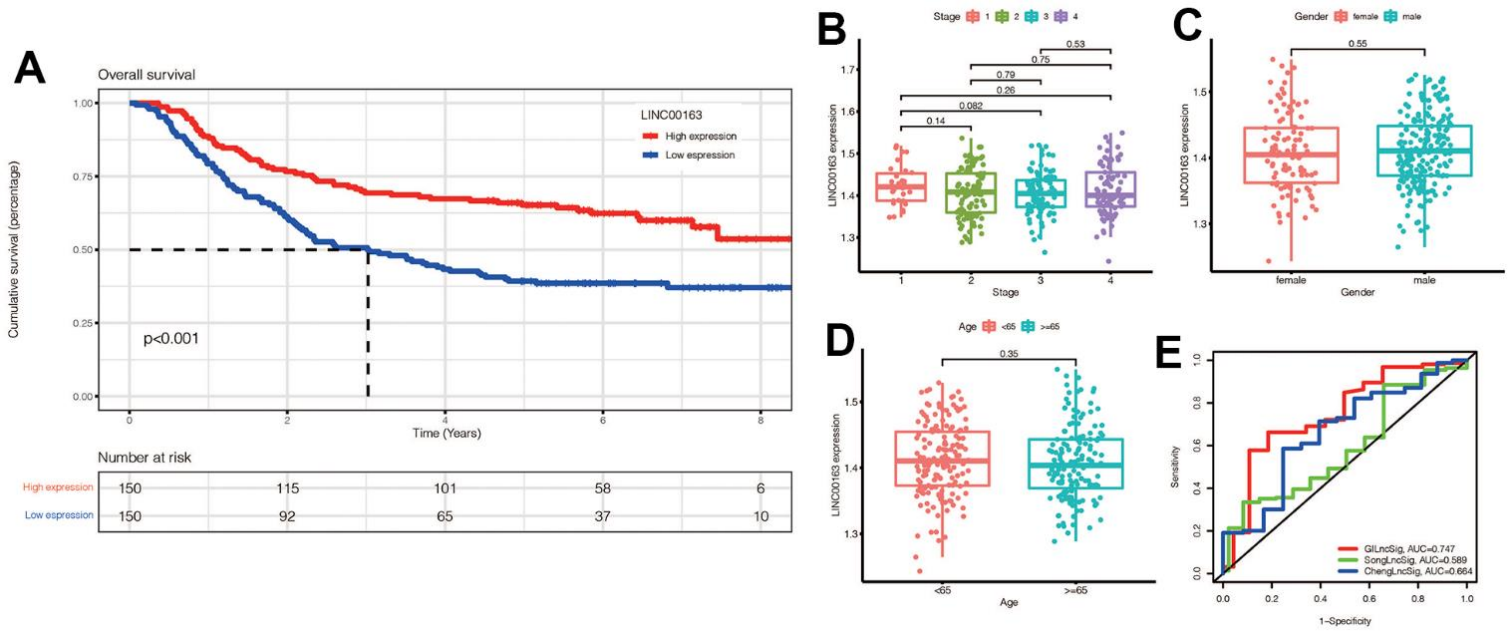
(**A**) KM survival curve of LINC00163 in gastric cancer:High expression of LINC00163 was associated with poor prognosis (*p*<0.001). (**B**–**D**) Expression of LINC00163 in different stages, genders and ages of gastric cancer. (**E**) Our model was compared with other models by plotting a 5-year ROC curve associated with prognosis.

We investigated the role of LINC00163 in the GSE62254 dataset and found that it was associated with prognosis, as shown in Figure 12A. Specifically, up-regulated LINC00163 expression was associated with a good prognosis in gastric cancer. This finding is consistent with experimental studies, which have shown that up-regulated LINC00163 can suppress gastric carcinoma development [26]. In our signature, the coefficient of LINC00163 was -0.15732, indicating that high expression of LINC00163 may result in a low-risk score and a good prognosis. As shown in Figure 12B–12D, we found that LINC00163 expression was similar across different gender, age, and stage groups.

We also compared our signature to other models, such as ChengLncRNAs and SongLncRNAs, and found that our model had a better prognostic value, with an area under the curve of 0.747 for 5-year prognosis (Figure 12E). This suggests that our signature may be a useful tool for diagnosing prognosis in gastric cancer patients [27, 28].

### PCR to verify the expression of LINC00163 in GC

**Figure 13:**
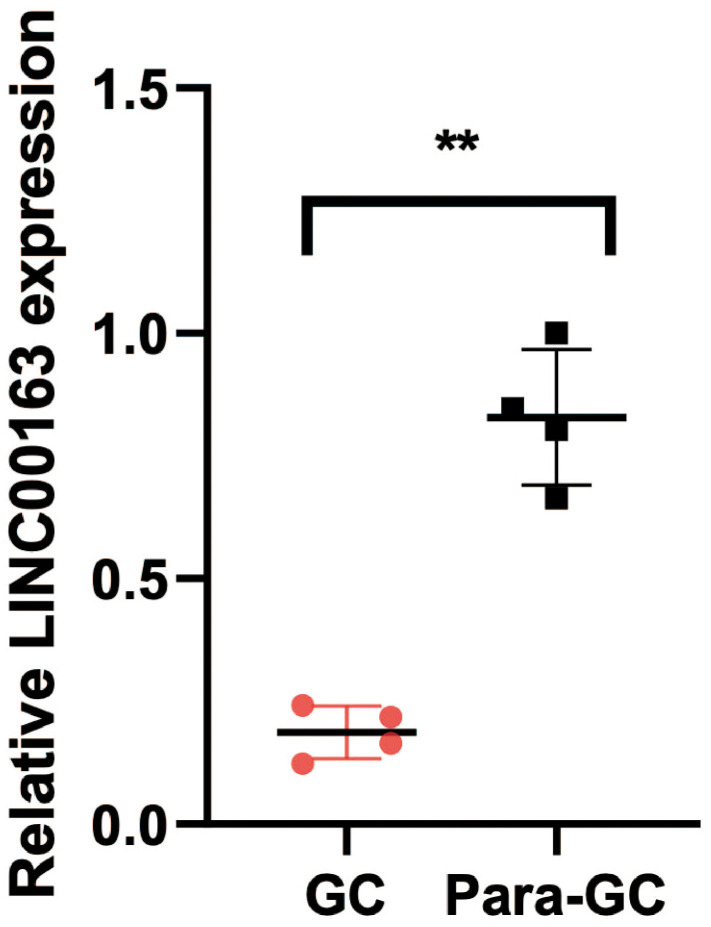
**PCR showed that LINC00163 was significantly up-regulated in 4 adjacent normal tissues compared with the GC tissues.** **p<0.01.

PCR was performed to verify the expression of LINC00163. The results showed that the expression of LINC00163 was significantly up-regulated in 4 adjacent normal tissues compared with the GC tissues. (Figure 13, **p<0.01).

## DISCUSSION

Genomic instability is a hallmark of cancer that underlies its heterogeneity and contributes to the phenotypes of many cancers [5]. In many cancers, there appears to be a threshold for genomic instability, below which increased genomic instability is usually associated with poor prognosis, while above this threshold, increased genomic instability is a favorable factor [5]. This raises the question of what mechanisms promote these phenomena, and whether tumors can be treated by altering their genomic instability according to “threshold properties”. Some researchers speculate that when the genomic instability of cancer exceeds the threshold, the immunogenicity of tumor cells increases and the replication process of cancer cells is affected by excessive gene mutations.

Gastric cancer is a highly heterogeneous tumor, and genomic instability is common in this cancer type [10]. Microsatellite instability-high tumors (MSI-H) have been recognized as a separate classification of gastric cancer, which is associated with a high degree of genomic instability due to the loss of function of the mismatch repair gene [29]. Interestingly, MSI-H tumors generally have a better prognosis than other types of gastric cancer and are often overexpressed with PDL-1, which allows them to benefit from immunotherapy [14]. This finding is consistent with the “threshold characteristics” of genomic instability, which may explain why excessive genomic instability can have a positive effect on tumor prognosis. Therefore, studying the prognostic value of genomic instability in gastric cancer is of great significance.

In this study, we developed a prognosis model for gastric cancer based on genomic instability-related lncRNAs. Our results showed that the higher-risk group had a worse prognosis, which was confirmed in the validation set. Additionally, Cox regression analysis demonstrated that the risk score was an independent prognostic indicator for gastric cancer. We also explored immune infiltration and immune checkpoints related to risk grouping, which could provide ideas for immunotherapy in gastric cancer. Finally, we identified drugs that were more sensitive in the high-risk group, which may offer potential benefits to patients with a poor prognosis. Collectively, our findings suggest that the genomic instability-related lncRNA signature has promising clinical implications for prognosis prediction, personalized treatment, and immunotherapy in gastric cancer.

Gastric cancer is often diagnosed in the late stages, when symptoms become more apparent, leading to poor prognosis [30–32]. Additionally, gastric cancer exhibits significant heterogeneity, further complicating its diagnosis and treatment. Therefore, developing a prognostic model for risk grouping can guide treatment and improve patient outcomes. Our study addressed this need by constructing a novel prognostic signature based on genomic instability-related lncRNAs, which provides valuable insight for the prognosis and treatment of gastric cancer.

Excessive activation of growth signals is associated with various cancers, including stomach cancer [33–35]. Functional and pathway enrichment analysis of the identified lncRNAs can reveal their main functions and signaling pathways, which is beneficial for our understanding of genomic instability. The results of our enrichment analysis provide references for crosstalks between genomic instability and critical biological functions and pathways that remain unclear. In particular, the cAMP signaling pathway and cell adhesion enriched by these lncRNAs have been associated with cancer [36–38].

The discovery of immune checkpoints is a milestone in the history of oncology therapy, greatly improving the survival of patients with advanced refractory tumors [39, 40]. The trend of immune checkpoint correlation was higher in high-risk patients, providing a reference for the development of treatment regimens based on risk scores. Immunotherapy, particularly with PD-1/PDL-1 inhibitors, has shown efficacy in immune-related tumors like melanoma. The higher correlation of immune checkpoints in high-risk groups suggests that immunotherapy may be more effective in these individuals, providing a potential therapeutic approach to improve outcomes.

Currently, several studies have constructed genomic instability related gene signatures in other tumor types. Zhu et al. constructed lncRNA signature with genomic instability to assess the prognosis and immune activity of patients with hepatocellular carcinoma (HCC). They found that the prognosis of high-risk patients was significantly worse, and the genomic instability score was related to chemotherapy drug sensitivity and immunotherapy effect of HCC patients [41]. Yang et al. also constructed a signature composed of five lncrnas to assess the prognosis and immune characteristics of patients with pancreatic ductal adenocarcinoma. They found that genomic instability features are associated with adaptive immunodeficiency immune profiles in pancreatic ductal adenocarcinoma and EMT and TME [42]. Thus, signature of genomic instability has potential value in prognostic stratification and immune evaluation of tumor patients. Our study can also provide some ideas for prognosis assessment of patients with gastric cancer.

There are two main types of genomic instability in gastric cancer: chromosomal instability (CIN) and microsatellite instability (MSI). CIN is characterized by a high frequency of chromosomal abnormalities, such as gains, losses, and rearrangements of chromosomes, while MSI results from defects in the DNA mismatch repair system, which leads to the accumulation of mutations in microsatellite regions of the genome.

Recent advances in genomic analysis have provided new insights into the molecular mechanisms underlying genomic instability in gastric cancer. For example, whole-genome sequencing studies have identified recurrent mutations in genes involved in the regulation of chromosome segregation, such as TP53, ARID1A, and CDH1, as well as in genes that control DNA damage response and repair, such as ATM, ATR, and CHEK2 [43].

Furthermore, studies have also shown that alterations in the expression and activity of certain enzymes involved in DNA repair, such as PARP1 and FEN1, can contribute to the development of genomic instability in gastric cancer. In addition, epigenetic modifications, such as DNA methylation and histone modifications, have been implicated in the regulation of genomic stability in gastric cancer [44–46].

LINC00163 is a long non-coding RNA that has been found to be dysregulated in several types of cancer. Its role in cancer appears to be context-specific, with studies showing it can act as both a tumor suppressor and an oncogene depending on the cancer type [47]. In our study, LINC00163 was found to be a key molecule in the signature of genomic instability. More research is needed to fully understand the mechanisms of action and potential therapeutic targeting of LINC00163 in cancer.

## CONCLUSIONS

The establishment of a prognostic model based on genomic instability can be of significance for the prognosis of gastric cancer patients. It is also meaningful for the exploration of genomics and treatment of gastric cancer patients. In the future, more high-quality studies are expected to reveal the mechanism and value of genomic instability in gastric cancer.
